# Eukaryotic ribosome quality control system: a potential therapeutic target for human diseases

**DOI:** 10.7150/ijbs.70955

**Published:** 2022-03-14

**Authors:** Peng-yue Zhao, Ren-qi Yao, Zi-cheng Zhang, Sheng-yu Zhu, Yu-xuan Li, Chao Ren, Xiao-hui Du, Yong-ming Yao

**Affiliations:** 1 Translational Medicine Research Center, Medical Innovation Research Division and Fourth Medical Center of the Chinese PLA General Hospital, Beijing, China.; 2 Department of General Surgery, First Medical Center of the Chinese PLA General Hospital, Beijing, China.; 3 Department of Burn Surgery, Changhai Hospital, Naval Medical University, Shanghai, China.; 4 Department of Orthopedics, Fourth Medical Center of the Chinese PLA General Hospital, Beijing, China.; 5 Department of Pulmonary and Critical Care Medicine, Beijing Chaoyang Hospital, Capital Medical University, Beijing, China.

**Keywords:** Ribosome, Ribosome quality control, Ribophagy, Ribosomopathy, Human diseases

## Abstract

Protein homeostasis is well accepted as the prerequisite for proper operation of various life activities. As the main apparatus of protein translation, ribosomes play an indispensable role in the maintenance of protein homeostasis. Nevertheless, upon stimulation of various internal and external factors, malfunction of ribosomes may be evident with the excessive production of aberrant proteins, accumulation of which can result in deleterious effects on cellular fate and even cell death. Ribosomopathies are characterized as a series of diseases caused by abnormalities of ribosomal compositions and functions. Correspondingly, cell evolves several ribosome quality control mechanisms in maintaining the quantity and quality of intracellular ribosomes, namely ribosome quality control system (RQCS). Of note, RQCS can tightly monitor the entire process from ribosome biogenesis to its degradation, with the capacity of coping with ribosomal dysfunction, including misassembled ribosomes and incorrectly synthesized ribosomal proteins. In the current literature review, we mainly introduce the RQCS and elaborate on the underlying pathogenesis of several ribosomopathies. With the in-depth understanding of ribosomal dysfunction and molecular basis of RQCS, therapeutic strategy by specifically targeting RQCS remains a promising option in treating patients with ribosomopathies and other ribosome-associated human diseases.

## Introduction

As the primary executors in the intracellular processes including growth, reproduction, and metabolism, proteins are essential components of almost all organisms 1. Thus, maintenance of protein homeostasis is the prerequisite for normal operation of life activities. Ribosome is known to be the main apparatus of protein synthesis, which is closely related to protein homeostasis 2. However, numerous stimuli can give rise to the production of aberrant proteins, including gene mutations, faults during expression of genes, chemical toxicant or the dearth of an interacting partner 3. Once aberrant proteins or truncated polypeptides are continuously accumulating, their deteriorative effects on cells may lead to cell death, thereby contributing to the development and progression of various human diseases 4, 5.

Recently, the ribosomal dysfunction has identified as one of the potential causes for abnormal protein production 6. Upon stimulation of internal and external factors, ribosomes may undergo profound malfunction and result in the production of abnormal proteins, which can disrupt cellular response, thereby contributing to the onset of several diseases. Many studies have documented that the alterations with respect to structure or function of ribosomal components cause a series of diseases, known as ribosomopathies, including Diamond-Blackfan anemia, 5q-syndrome, Schwachman-Diamond syndrome, Dyskeratosis Congenita, Cartilage hair hypoplasia, and Treacher Collins syndrome, the majority of which represent congenital diseases characterized by haploinsufficiency of genes encoding ribosomal proteins 7. However, as a stable organelle with a half-life of several days, ribosomes evolve mechanisms that are capable of degrading redundant or dysfunctional ribosomes and adjust numbers to the changing environment.

Ribosome quality control (RQC) refers to the process, by which eukaryotes monitor the stagnation and collision of ribosomes during protein synthesis, and effectively eliminate abnormal peptides. The monitoring of protein homeostasis and functioning ribosomes are not merely restricted in the process of protein synthesis, but it goes through the entire process from ribosome synthesis to degradation. Based on this theory, we are proposed the concept of ribosome quality control system (RQCS), which generally represents a self-protective system underlying regulation of the biogenesis, transportation, and degradation of the ribosomes upon exposure to unfavorable internal or external stimuli. Unlike the concept of RQC, RQCS primarily focuses on the quality control processes of the ribosome itself rather than the ribosome-associated protein synthesis process, which covers a broader scope that includes each step of ribosome quality control from biogenesis to degradation. Of note, RQCS play an important role in alleviating ribosomal dysfunction and restoring protein homeostasis 8. Taking autophagy as an example, it can engulf and degrade damaged or excessive organelles and intracellular components, in turn maintaining and restoring the intracellular homeostasis. Likely, ribosome-specific autophagy, a highly selective type of autophagy, namely ribopahgy, can effectively yet specifically degrade damaged or excessive ribosomes. Therefore, ribophagy is deemed as a key player in quality control of ribosomes, and it is one of the potential mechanisms concerning RQCS.

**Figure 1:**
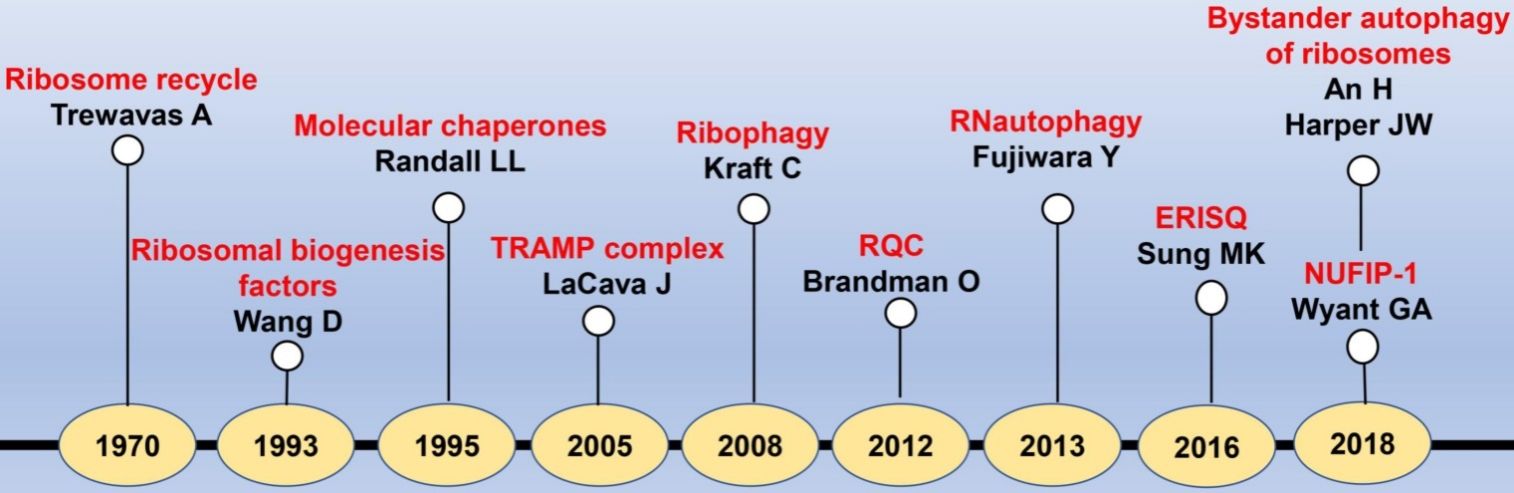
** The timeline diagram of major events in the discovery of RQCS.** Abbreviation: TRAMP: TRf4/5-Air1/2-Mtr4 polyadenylation; RQC: ribosome quality control; ERISQ: excess ribosomal protein quality control; NUFIP-1: nuclear fragile X mental retardation-interacting protein 1.

To the best of our knowledge, there are several reviews regarding to the ribosome-associated protein quality controls, while the quality control process of ribosome itself has been rarely reported^9^, ^10^. In this review, we mainly introduced the RQCS from the generation of ribosome to its degradation (**Fig. 1**). Moreover, we comprehensively discussed the ribosome related diseases, for which we illustrated the potential pathogenesis at the molecular level, hopefully providing novel curative targets for the handling of various ribosomopathies.

## The components, distribution, and functions of ribosomes

### The components of ribosomes

Ribosome represents an elaborately assembled organelle consisting of ribosomal RNA (rRNA), ribosomal proteins (RPs), and small nucleolar RNAs (snoRNAs) 11. In addition to being an essential part in forming ribosome, each component plays independent yet multifunctional role. For example, rRNA mainly functions as scaffold catalyzing the formation of peptide bond during protein synthesis, whereas RPs are responsible for optimizing rRNA processing and stabilizing the ribosomal structure. Meanwhile, snoRNAs primarily regulate the chemical modifications of ribosomes 12. Macroscopically, eukaryotic 80S ribosomes comprise the 60S large subunit (LSU) and the 40S small subunit (SSU), in which the former is about twice the size of the latter 13. Notably, the subunits of different species are not identical in light of the compositions. Specifically, SSU of eukaryotes are composed of 18S rRNA and 33 different RPs, while LSU contains 5S rRNA, 5.8S rRNA, 25S-28S rRNA, and 46 (in yeast)/47 (in human) RPs. Functionally, LSU mainly catalyzes formation of peptide bond with the peptidyl transferase center (PTC), whereas SSU serves as the decoding center to bring together messenger RNAs (mRNAs) and aminoacyl-transferRNAs (tRNAs) in order to translate the genetic code 14.

### The distribution of ribosomes

As we know, ribosomes mainly locate in the cytoplasm. Corresponding to the subcellular localization of ribosomes, we categorize them into free ribosomes and attached ribosomes, with the former is the main executor of translation. In addition to cytoplasm, ribosomes can present in the mitochondrion, which are characterized by mitoribosomes 15. Mitoribosomes primarily locate in the matrix of organelles and specialize in synthesis of a handful of polypeptides that are essential in facilitating ATP production aerobically and oxidative phosphorylation (OXPHOS) 16.

### The function of ribosomes: translation

Translation is deemed as a precisely regulated process, in which ribosomes serve as the most significant executor and platform during this process. Based on the features across disparate stages of translation, it can be divided into four steps: initiation, prolongation, termination, and ribosome recycling 17. Ribosomes play different roles in each step throughout the translational process 18. At the initial stage of translation, the LSU and SSU of eukaryotic ribosomes are separated upon the action of initiation factor eukaryotic initiation factor 3 (EIF3), followed by formation of the translation initiation complex containing SSU combined with mRNA and Met-tRNAi. During the elongation step, each aminoacyl-tRNA completes the entrance, peptide band formation and translocation by binding to the A, P, and E sites on the SSU in sequence. By the time that ribosome encounters a termination codon, the peptidyl-tRNA linkage is hydrolyzed to release the nascent peptide chain, and the mRNA is separated from ribosome with the aid of release factors. Finally, detached LSU and SSU are reassembled to form ribosomes in the cytoplasm prior to circulation of the above translational process 19, 20.

## Ribosomal dysfunction

Ribosomal dysfunction refers to the loss of physiological function of ribosomes due to various factors, including lack of ribosomal components, alterations in the ribosomal structures, and disturbances of the ribosomes during the translational process. Numerous studies have shown that the incidence of ribosomal dysfunction is significantly increased upon starvation, nitrogen deficiency, mammalian target of rapamycin (mTOR) inhibition, and ultraviolet radiation 21, 22. The recent study has been proposed that the golden standard for evaluating ribosomal dysfunction can be ribosome collision 23. Ribosome collision is defined as a special state when ribosome stalls on the mRNA and collides with the latter one during translational process 24. Ribosome collision is commonly caused by ribosome stagnation, which can be further categorized into monosome, disome, and trisome based on the number of ribosome collision. Correspondingly, the occurrence of ribosome collision can be preliminarily determined by the presence of disome and trisome. Currently, there are two well-established methods to evaluate ribosome collision, for which the first one is ribosome profiling, and the second one is to separate samples with a sucrose gradient of 10% to 50%, followed by immunoblotting of representative ribosome subunits (ul2 and eS24). These collided ribosomes need to be degraded in time, otherwise their accumulations might result in generation of misfolded proteins and induction of proteotoxic stress, thereby compromising cell survival 25. In addition, collided ribosomes affect the subsequent translational process 26.

Notably, ribosomal dysfunction can occur throughout the entire processes, from the synthesis of ribosomal components, the assembly and transportation of ribosomal subunit precursors, to the degradation and autophagy of ribosomes. Fortunately, as a precision-assembled organelle, ribosome evolves a variety of RQCS to counter and alleviate ribosomal dysfunction. Nevertheless, the malfunction and dysregulation of RQCS obviously contribute to the ribosomal dysfunction. Next, we will elaborate on the RQCS from the aspects of ribosomal biogenesis, transportation, degradation, and autophagy.

## Ribosome quality control system

### Ribosomal biogenesis

**Figure 2:**
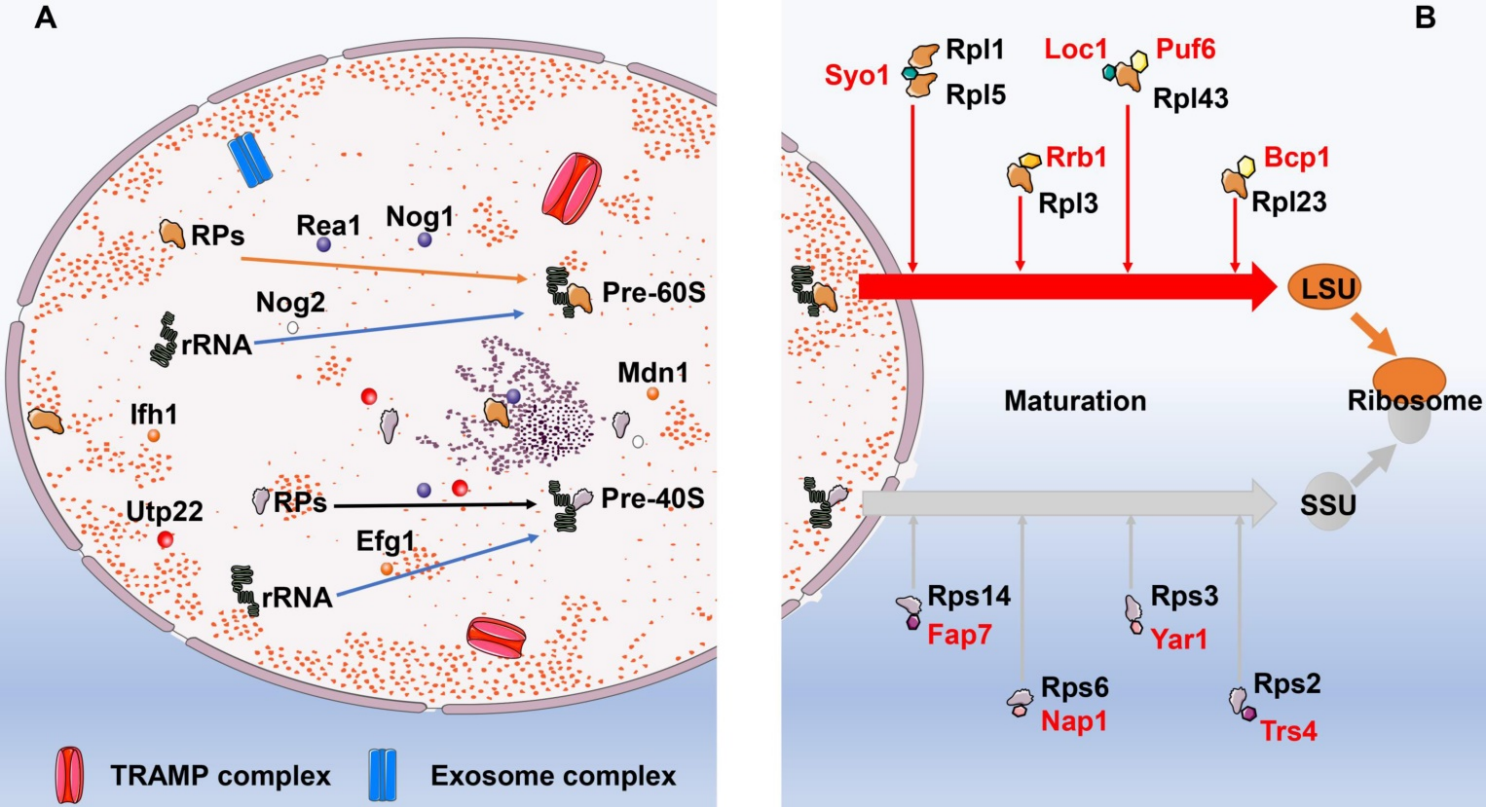
** The biogenesis and maturation of ribosomes. A:** The biogenesis of ribosomes. In the nucleolus, ribosomal proteins firstly are combined with pre-ribosome RNAs (pre-rRNA) to form pre-60S and pre-40S. This process is regulated by multiple molecules and dedicated chaperones. These essential biogenesis factors include Ifh1, Utp22, U3-snoRNP, UTP-A, UTP-B, and UTP-C. In addition to above mentioned molecules, a number of complexes are manifested to play roles in the surveillance and turnover of misassembled preribosomes as well, such as the TRAMP complex and exosome complex of exonucleases. **B:** The maturation of ribosomes. Following synthesis of ribosomal subunit precursors in the nucleus, they need to enter the cytoplasm via the nuclear pore prior to combining with RPs to complete the maturation process. Dedicated chaperones specifically transport RPs to ensure the successful implementation of this process. The dedicated chaperones in the biogenesis of 60S LSU include Rrb1, Sqt1, Acl4, and Bcp1. While the dedicated chaperones Fap7, Nap1, Yar1, and Trs4 are involved in the maturation of 40S SSU. Abbreviation: Ifh1: interacts with forkhead 1; Utp22: U3 small nucleolar RNA-associated protein 22; UTP: U3 small nucleolar ribonucleoprotein particles; TRAMP: TRf4/5-Air1/2-Mtr4 polyadenylation; RPs: ribosomal proteins; LSU: the 60S large subunit; SSU: the 40S small subunit.

Ribosomal biogenesis initiates at the nucleolus, where RPs are associated with pre-ribosome RNAs (pre-rRNA) cotranscriptionally 27. Thereafter, the preribosomal particles travel from nucleolus to the cytoplasm, where they ultimately mature into translation-competent ribosomal subunits. Corresponding to the complexity and error-prone of this process, ribosome biogenesis is elaborately regulated by multiple signaling pathways as well as dedicated chaperones in response to increased protein demand or recovery of ribosomal network 28. These essential biogenesis factors can monitor and timely correct errors occurred during the assembling process to ensure the biogenesis of functional ribosomes, including ribosome protein gene transcriptional activator interacts with forkhead 1 (Ifh1), the rRNA processing factor U3 small nucleolar RNA-associated protein 22 (Utp22), U3 small nucleolar ribonucleoprotein particles (U3-snoRNP), U three-associated protein (UTP)-A, UTP-B, and UTP-C 29-31. For example, Exit of G1 (Efg1) was a novel yet pivotal surveilling molecule of pre-40S particles in *Saccharomyces cerevisiae* other than ATPase Rio1 and Rio2, for which the specific mechanism was that Efg1 assisted 11S RNA targeting to the TRf4/5-Air1/2-Mtr4 polyadenylation (TRAMP) complex. Similarly, Rea1 and Nog2 were reportedly involved in sensing the correctly assembled status of pre-60S ribosomes and triggering remodeling events. Moreover, the GTPase Nog1 could occupy the peptide exit tunnel and act as the final monitor prior to Pre-60S particles leaving the nucleolus 32. Likewise, Chen and his colleagues found that Midasin AAA ATPase 1 (Mdn1) was a significant ATPase, not only associated with various activities that required for terminating ribosome biogenesis, but also removed assembly factors from distinct precursors of the ribosomal 60S subunit 33. In addition to above mentioned molecules, a number of complexes are manifested to play roles in the surveillance and turnover of misassembled preribosomes as well, such as the TRAMP complex and exosome complex of exonucleases 34 **(Fig. 2)**. TRAMP allegedly targets anomalous or less steady tRNA by polyadenylating the 3′ end to furnish a single-stranded RNA extension that is long enough to facilitate the engagement of the Mtr4 helicase 35. Das et al. 36 found that exoribonuclease activities of Rrp6-associated RNA exosomes protected unfluctuating RNAs from TRAMP-mediated polyadenylation and degradation, whereas the catalytic activity of the exosome-TRAMP complex contributed to substrate distinction and degradation of less steady RNAs.

### Ribosomal protein transportation

Following synthesis of ribosomal subunit precursors in the nucleus, they need to enter the cytoplasm via the nuclear pore prior to combining with RPs to complete the maturation process. Gerhardy and his colleagues reported that the low temperature induced assembly protein, Puf6, primed 60S pre-ribosome nuclear exportation and intervened rRNA compaction, was a momentous temperature-regulated rescuing mechanism underlying countering rRNA misfolding and priming exportation competence 37. Meanwhile, dedicated chaperones specifically mediate the transportation of RPs to ensure the successful implementation of this process. Recently, study by Liang et al. 38 found that both Puf6 and Loc1 were the dedicated chaperones of ribosomal protein Rpl43, which could form a ternary complex required in biogenesis of 60S LSU. Similarly, Black et al. 39 reported that Tsr4 was a cytoplasmic chaperone dedicated to Rps2; Rossler et al. 40 carried out a tandem-affinity purification-based screen and identified Nap1 and Tsr4 as direct binding partners of Rps6 and Rps2, respectively. In addition to newly discovered molecular chaperones, Rrb1, Sqt1, Acl4, and Bcp1 have long been demonstrated to be the dedicated chaperones of the large ribosomal subunit Rpl3, Rpl10, Rpl4, and Rpl23 41-45. Syo1 acts as a chaperone that binds two 60S r-proteins Rpl5 and Rpl1 at contemporaneously 46, whereas Yar1,Tsr2, and Fap7 are dedicated chaperones of the 40S subunit r-proteins Rps3, Rps26, and Rps14, respectively 47-50 **(Supplemental Table 1)**.

### Ribosomal degradation

#### Ribosome recycle

Ribosome stagnation is the initial stage of ribosome cycle. A variety of physical and chemical stimuli can lead to the occurrence of ribosome stagnation, including two arginine CGA codons contiguous to each other, numerous proline codons arrayed in a row, chemical detriment, endonuclease activity, and mistakes in gene expression. It has been well studied that the mRNAs and abnormal proteins produced by ribosome stagnation can be degraded by no-go decay (NGD) and RQC pathways, respectively 51. Thus, how to deal with the collided ribosomes produced in the translational process should be taken into consideration. Correspondingly, it brings about the conception of “ribosome recycle”, for which the collided ribosomes are separated into large and small subunits followed by re-participation into the translational process.

**Figure 3:**
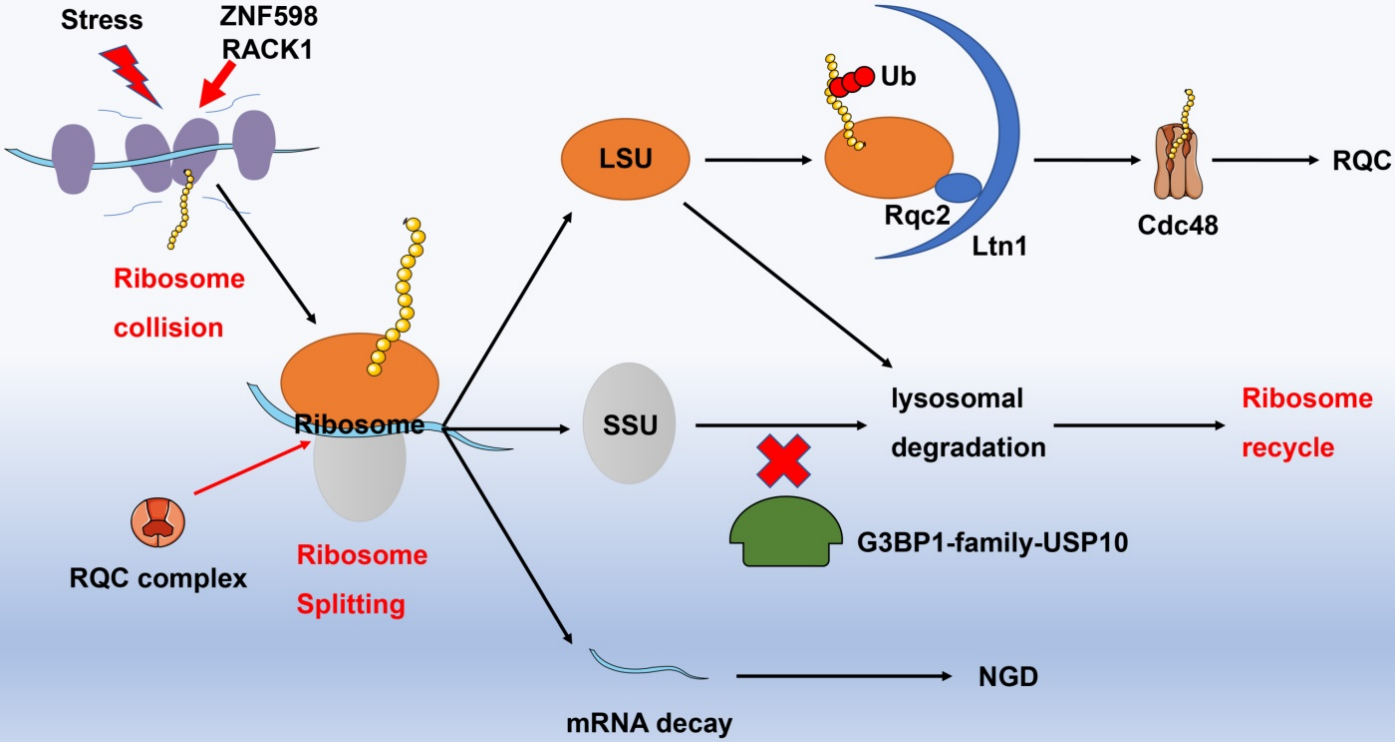
** Ribosome recycle.** Under stress, adjacent ribosomes performed protein translational function may collide. ZNF598 and RACK1 can identify colliding ribosomes, and recruit RQC complexes to split the colliding ribosomes. The splitting mRNA strands enter the NGD degradation pathway; the splitting large ribosomal subunits are specifically recognized by Ltn1 and Rqc2, while the semi-synthetic polypeptide chain is labeled by ubiquitinated proteins and then degraded into amino acids under the action of Cdc48, in terms of RQC process. A part of the splitting small ribosomal subunit is directly degraded by lysosomes (blockade with G3BP1-family-USP10), but another part that escapes lysosomal degradation recombines with the large ribosomal subunits and enters into the ribosomal recycle to perform protein translation functions. Abbreviation: RACK1: receptor for activated C kinase 1; RQC: ribosome quality control; NGD: no-go decay.

Recognition of collided ribosomes and separation of 40S as well as 60S subunits are initial steps of ribosome recycle. The latest research has indicated that E3 ligase ZNF598 in mammalian cells (Hel2 in yeast) recognizes the interface of 40S unit of collided ribosome with the aid of receptor for activated C kinase 1 (RACK1, Asc1 in yeast) 52, 53. The ribosome subunits are then labeled by ubiquitin and dissociated involving Dom34, Hbs1, Rli1 (PELO, HBSL1, ABCE1 in higher eukaryotes), and RQC complexes. For instance, Dom34 removes stalled ribosomes from truncated mRNAs and rescues ribosomes in 3'untranslated regions 54, while the GTPase Hbs1 senses the ribosome stagnation in budding yeast and load Dom34 55. Likely, the ATPase Rli1 disassembles the 80S-like complexes composed of large (60S) subunits and pre-40S subunit 56. The 60S subunit incorporated peptidyl-tRNA is further handled by NEMF and LTN1, which are capable of polyubiquitinating the polypeptide 57. In addition, G3BP1-family-USP10 complexes can rescue ubiquitinated 40S subunits by preventing them from lysosomal degradation. With regard to the relevant mechanism, USP10 might deubiquitinate components of the ribosomal 40S SSU, including RPS2, RPS3, and RPS10. Ribosomal subunits escaped from lysosomal degradation subsequently re-assemble and enter the ribosome recycle for exerting protein translational functions 58 **(Fig. 3)**.

#### Excess ribosomal protein degradation

**Figure 4:**
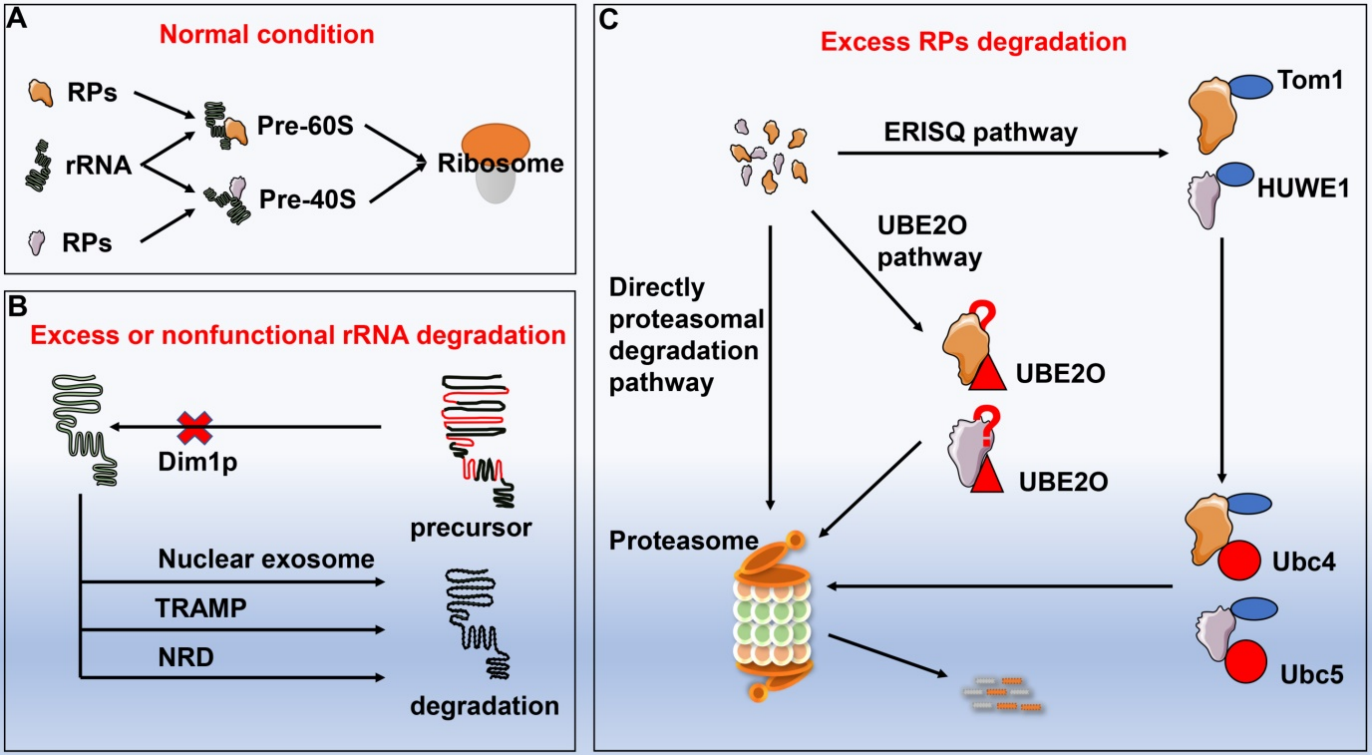
** The degradation of excessive or non-functional ribosome proteins and rRNA. A:** Ribosomes are assembled by rRNA and RPs following strict proportion. If certain stimuli impair ribosome synthesis process, RPs or rRNA will be overproduced disproportionately. **B:** The excessive or non-functional rRNA degradation pathways include Dim1p pathway, nuclear exosome pathway, TRAMP pathway, and NRD pathway. **C:** The excessive RPs degradation pathways include directly proteasomal degradation pathway, ERISQ pathway, and UBE2O pathway. Abbreviation: RPs: ribosomal proteins; TRAMP: TRf4/5-Air1/2-Mtr4 polyadenylation; NRD: nonfunctional rRNA decay; ERISQ: excess ribosomal protein quality control; UBE2O: ubiquitin conjugating enzyme E2O.

Ribosomes are assembled from rRNA and RPs with proper proportion. Impairment of rRNA synthesis by genetic mutations or thallium treatment can result in disproportionately generated RPs. Excessive RPs are needed to be degraded in time, otherwise they might lead to proteotoxic stress. However, no substantial progress has been made on the underlying mechanisms with respect to degradation of redundant RPs until the discovery of ubiquitin-proteasome system. Sung et al.^59^ reported that inhibition of the proteasome induced the accumulation of multiple endogenous ribosomal proteins within insoluble aggregates, implicating overproduced RPs were rapidly ubiquitinated and degraded through a proteasome-dependent manner. Furthermore, redundant RPs yielded by transcriptional or translational imbalance were initially ubiquitinated by enzyme named Tom1 (yeast) or HECT, UBA, and WWE domain containing E3 ubiquitin protein ligase 1 (HUWE1, human) and subsequently collaborated with the E2 enzymes Ubc4 and Ubc5, followed by entering the proteasomal degradation process. Unlike autophagy that is capable of degrading entire ribosome, ubiquitin-proteasome system mainly degrades individual RP, which is subjected to the excess ribosomal protein quality control (ERISQ). In addition to the ERISQ pathway, there are another two pathways that control the turnover of excess RPs based on their sources. Excess RPs generated from chromosomes can be degraded by the proteasome directly, while redundant ribosomal subunits yielded during erythroid development are eliminated via the ubiquitin conjugating enzyme E2O (UBE2O) 60. Nevertheless, how UBE2O selected specific ribosomal proteins for degradation remains further elusive 61 **(Fig. 4)**.

#### Excessive or nonfunctional rRNA degradation

Similar to the RPs, excessive or nonfunctional rRNAs need to be eliminated, otherwise their accumulations in turn hinder the translational process 62. Lafontaine and his colleagues noted that Dim1p impeded the maturation of the precursor rRNA in the absence of demethylation, which was deemed as the firstly discovered quality-control pathway of rRNA 63. Hence, several pre-rRNA quality control pathways have been documented, including the nuclear exosome pathway in degrading nuclear-restricted rRNAs and the TRAMP complex-mediated pathway 64-67. LaRiviere et al. 68 identified a novel rRNA quality-control mechanism regarding to the degradation of the excessive or nonfunctional rRNA components of ribosome, commonly known as nonfunctional rRNA decay (NRD). Unlike the above quality-control pathways for degradation of precursor rRNA, NRD can specifically target mature 18SrRNA and 25SrRNA. Of note, 18S NRD exclusively occurs in the cytoplasm, whereas 25S NRD can be initiated in both cytoplasm and nucleus with unknown mechanisms.

### Autophagy

Autophagy represents a highly conserved process, and it encapsulates the macromolecules and impaired organelles by forming a double-layer membrane followed by transporting them to lysosomes for degradation. Recent studies have demonstrated that autophagic degradation of ribosomes upon starvation includes three pathways: non-selective autophagy, endoplasmic reticulum autophagy (ER-phagy) bypass, and receptor-mediated selective autophagy (ribophagy) 69, 70.

#### Nonselective autophagy of ribosomes

Currently, numerous studies suggest that autophagy can act as a quality control manner in degrading excessive or impaired organelles, thereby maintaining homeostasis of organelles under the challenge of various internal or external stimuli 71-73. For example, autophagy is proven to restore modification and processing function of endoplasmic reticulum by resolving endoplasmic reticulum stress in the setting of septic insults 74. Likewise, upon starvation or energy deprivation, autophagy degrades dysfunctional mitochondria to ensure the well-organization of respiratory chain and synthesis of ATP 73, 74. As the pivotal organelle for protein translation, recent reports have indicated that superfluous or damaged ribosomes can be degraded by non-selective autophagy in various conditions, including proteotoxic stress and nutrition deficiency 69. Therefore, autophagy is thought to be one of significant mechanisms for maintaining the homeostasis between ribosome biogenesis and degradation.

Nevertheless, whether autophagy served as a mechanism concerning RQC has long been controversial. Previous proteome research showed that the ribosome abundance was unaffected when knocking out *ATG5* gene in mouse embryonic fibroblasts in starvation 75. Quantitative analysis of fibroblasts revealed an identical conclusion, in which half-life of ribosomal protein RPL11 was not significantly altered when silencing the key autophagy-related genes, including *ATG5* and *ATG7* 76, 77. These experiments indicated an unexpected finding that regulation of ribosome abundance upon starvation was not associated with autophagic induction. However, recent study that employed the Keima protein fused with ribosomal proteins RPL28 and RPS3 in measuring actual autophagic flux, brought about a completely divergent conclusion 78. An et al. 98 confirmed that Ribo-Keima flux in lysosomes was significantly augmented in response to starvation or inhibition of mTOR. The activation of Ribo-Keima flux in HEK293 cells and HCT116 cells was VPS34-dependent, which was also a key PI3P kinase involving in canonical autophagy. Interestingly, when knocked out the *ATG5*, ribosome autophagy was not prominently influenced. On the one hand, autophagy accounts for degradation of only a small part of ribosomes; on the other hand, the intensity of Ribo-Keima flux is not solely determined by autophagosomes. Thus, total autophagy flux might be attributed to any vesicular structure encapsulating the ribosomes for lysosomal degradation.

With the gradual popularization of the monitoring of autophagic flux, although autophagy contributes to the degradation of a fraction of ribosomes and is usually mobilized under cellular stress, increasing evidence has been demonstrated that autophagy is one of the significant pathways for restraining ribosome abundance in recent years 2, 69, 79. For example, dysfunctional ribosomes are focused for autophagic degradation to sustain the quality of ribosome population. Especially when the cells encounter various stresses, including hunger, drug toxicity or other stimuli, the proteins will be temporarily deficient, the ribosomes are plentifully assembled to meet the elevated demand of protein translation. Since ribosomes may be misassembled or disproportionately over-generated during this process, autophagy will act as a RQC pathway to degrade nonfunctional or redundant ribosomes.

#### Selective ribosome autophagy-ribophagy

In addition to non-selective autophagy, whether ribosomes possessed selectively autophagic pathway like the other organelles such as endoplasmic reticulum and mitochondria have been inconclusive. It is widely accepted that if a substance or organelle has a selective degrading pathway, it includes specific receptors that not only bind the autophagosome, but selectively recognize the substance or organelle needed to be degraded. Notably, Gregory's team made a groundbreaking discovery, in which they firstly determined nuclear fragile X mental retardation-interacting protein 1 (NUFIP-1) as a specific receptor for famine-induced ribophagy in mammal. This study potently confirms the existence of selective autophagy targeting ribosomes and provides new insights into the study of ribophagy 80.

The concept of ribosomal autophagy was initially proposed by Kraft et al. 81 in *Saccharomyces cerevisiae*, in which they found that mature ribosomes were degraded by both non-selective autophagy as well as a novel selective pathway upon nutrient starvation, namely 'ribophagy'. Moreover, they reported the autophagic degradation of mature ribosomes was closely related to the ubiquitination pathways. Interestingly, Matthias and his colleagues demonstrated that the UBp3-Bre5 deubiquitylation complex could inhibit mitophagy, reflecting the complexity of regulation with regard to selective autophagy and ubiquitination 82. Ossareh-Nazari and his team found that Cdc48 and Ufd3 served as the major executor of ubiquitin proteasome system, and they were indispensable for Ubp3-Bre5-dependent and starvation-induced selective autophagy of mature ribosomes in *Saccharomyces cerevisiae*, since these molecules worked synergistically in recognizing and deubiquitinating substrates of ribophagy specifically 83. Of note, Ltn1 was identified as a critical player in mRNA surveillance and ribosome-associated quality control, which could antagonize Ubp3 and protect ribosomal 60S subunit from starvation-induced selective autophagy 84.

Considering the lack of specific receptors for selective autophagy targeting ribosomes and molecular structure of ribosomes, the relevant research on ribophagy has been progressing slowly and majority of studies focus on yeast. By applying* Saccharomyces cerevisiae*, a study conducted by Tatehashi et al. 85 revealed that γ-glutamine kinase could interact with Ubp3 and participate in ribophagy other than involving in the biosynthesis of proline. Ribophagy was obviously blocked when suppressing the expression of γ-glutamine kinase via knocking out its encoding gene *PRO1*, indicating its importance in ribophagy induction 85. Meanwhile, downstream kinases Sch9 and Rim15 of mTOR complex 1 (mTORC1) was demonstrated to play an essential role in the autophagic degradation of ribosomes in budding yeast 86. Intriguingly, after inactivation of mTORC1, Rim15 downregulated non-selective ribosomal degradation, whereas upregulated ribophagy. Therefore, researchers speculate a model that Rim15 might modulate the equilibrium between selective and non-selective degradation of ribosomes.

Pioneering work by Gregory's team firstly identified NUFIP-1 as the specific receptor for starvation-induced ribophagy in mammal. Notably, NUFIP-1 could not only bind the ribosomal 60S large subunit under the help of Zinc finger hit domain-containing protein 3 (ZNHIT3), but it possessed 4 domains that interacted with LC-3B as well, namely LIR, through which NUFIP-1 was capable of transporting the ribosomal 60S large subunit to autophagosomes for degradation. These results suggested that NUFIP-1 mediated ribophagy provided host with necessary metabolites that were indispensable for cell survival upon starvation, indicating that ribophagy was one of the important quality control processes in maintaining protein homeostasis. However, the precise mechanism for degrading small ribosomal subunit requires in-depth investigation.

Since the role of NUFIP-1 as a specific receptor for ribophagy has been identified, other functions of NUFIP-1 have gradually attracted attention globally 87. Intriguingly, Shim et al. 88 found that NUFIP-1 could also translocate from the nucleus to lysosomal associated membrane protein 2 (LAMP2) under cyclic mechanical stress without triggering ribophagy, hinting a more general effect of NUFIP-1 as a receptor for yet-to-be-identified targets in cyclic mechanical stress and as a molecule for the surveillance of nuclear LC-3 against stretch-induced damage, other than acting as a synthesizer of snoRNP and receptor for ribophagy.

#### By-stander flux during selective autophagy of organelles

**Figure 5:**
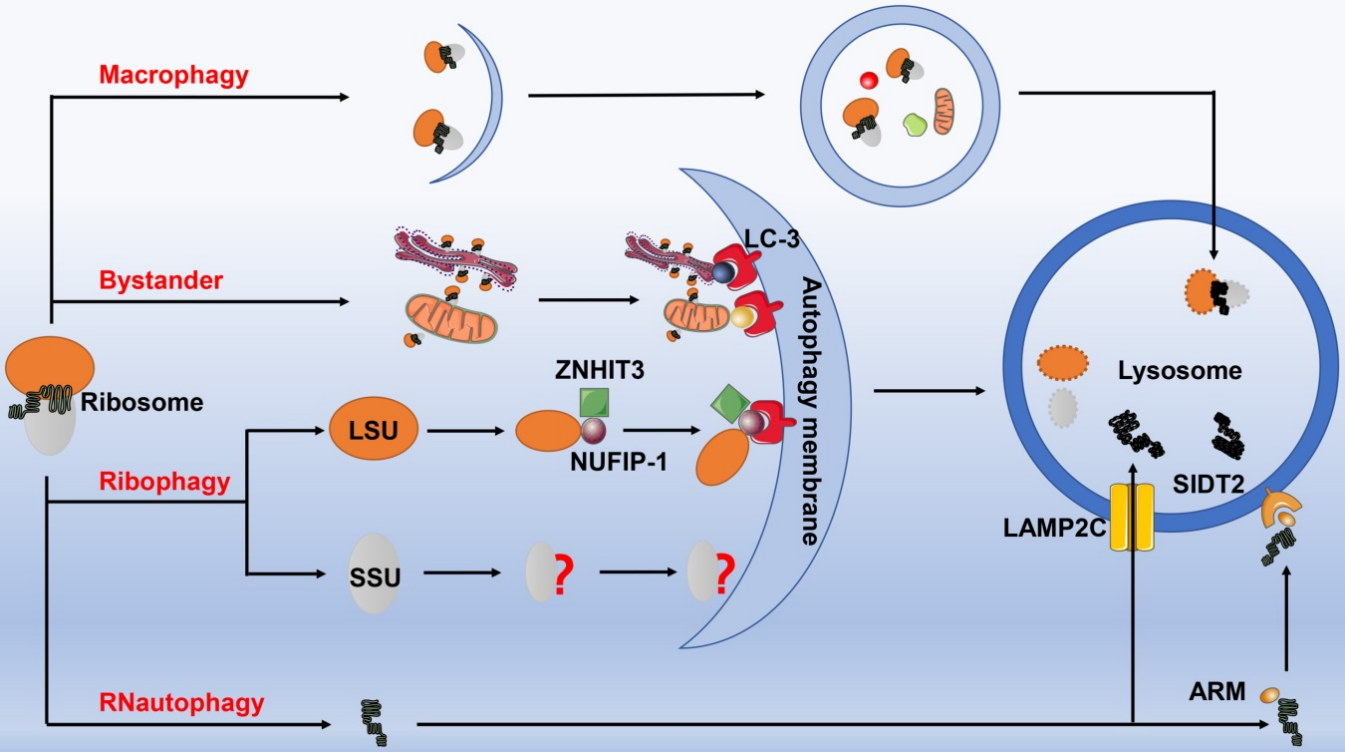
** The autophagy of ribosomes.** Ribosomes can be degraded by four autophagic pathways. 1) Macrophagy. Ribosomes are non-selectively wrapped by autophagosomes, and then fused with lysosomes to form autophagic lysosomes for degradation. 2) Ribophagy. The ribophagy receptor NUFIP-1 specifically recognizes the large ribosomal subunits under the help of ZNHIT3, and then binds to the LC-3B on the autophagosome membrane to complete the next process, however, the precise mechanism for degrading small ribosomal subunit remains to be elucidated. 3) Bystander autophagy pathway. When other selectively organelle autophagy occurs, such as mitochondrial autophagy, endoplasmic reticulum autophagy, etc., ribosomes can be engulfed and degraded incidentally. 4) RNautophagy. rRNA directly enters the lysosome for degradation through the protein receptor LAMP2C and SIDT2 (with the assistance of ARM) on the lysosomal membrane. Abbreviation: NUFIP-1: nuclear fragile X mental retardation-interacting protein 1; ZNHIT3: Zinc finger HIT domain containing protein 3; LAMP2C: lysosomal associated membrane protein 2C; SIDT2: SID-1 transmembrane family member 2; ARM: arginine-rich motif.

The synergy of the structure and function of various organelles is the prerequisites for intracellular homeostasis. As a key organelle for protein synthesis, ribosome is structurally and functionally correlated with other organelles, including endoplasmic reticulum and mitochondria. Recent studies have shown that there are intricate relationships across various organelles in the selective autophagy of organelles. An and Harper unexpectedly discovered that a fraction of ribosomes could be packaged and degraded incidentally during the process of selective autophagy targeting other organelles 78. Although the ratio was relatively low, it revealed the potential relationship among disparate types of organelle-specific autophagy, and pioneered the research on another pathway of ribosomal autophagy, in terms of “bystander” autophagy. A recent study conducted by the same team manifested that some ribosomes were specifically degraded along with ER-phagy, in favoring of their previous findings^89^ **(Fig. 5)**.

#### RNautophagy: direct uptake of nucleic acids by lysosomes

Fujiwara and his colleagues reported a novel autophagic pathway specifically degrading rRNA, known as RNautophagy 90. Identical to the chaperone-mediated autophagy (CMA), RNautophagy also directly transports RNA to the lysosomes for degradation. This process requires ATP for providing energy, in which lysosome-associated membrane glycoprotein 2C (LAMP2C) serves as a specific receptor. Unlike CMA, heat shock 70 kD protein 8 (HSPA8) exerts no marked impact on the uptake of RNA into the sequestered lysosomes. In addition, LAMP2C can bind all types of RNAs, and RNautophagy is responsible for the degradation of estimated 10%-20% of the total amount of RNA in living cells. However, RNautophagy possesses selectivity regarding to RNA substrates, for which substrates containing poly-G/dG or at least some consecutive G/dG sequences are recognized and mobilized to the cytosolic region of LAMP2C 91. Thereafter, another RNautophagy receptor contributed to lysosomal uptake and degradation of nucleic acids has been identified, namely SID-1 transmembrane family member 2 (SIDT2), the ortholog of the *Caenorhabditis elegans* putative RNA transporter SID-1^92^. Of note, approximately half of the RNA degradation was inhibited at the cellular level by knocking down *SIDT2* gene. Intriguingly, the two receptors SIDT2 and LAMP2C were proven to work independently. Although the protein-protein interaction has been identified between these two molecules, SIDT2 overexpression potentiates RNA uptake in the absence of LAMP2C. The latest research has confirmed that SIDT2 directly binds RNA and DNA via an arginine-rich motif (ARM), destruction of which substantially weakens SIDT2-mediated RNautophagic activity 93 **(Fig. 5)**.

## Ribosomal dysfunction and human diseases

Ribosomopathies represent a series of diseases caused by ribosomal dysfunction, including structural and functional abnormality as well as the alteration in the compositions of ribosomes. A variety of ribosome-related diseases have been reported, including congenital dysplasia diseases, hematological diseases, neurological diseases, malignant tumors, and diseases of other systems 94. Although the underlying pathogenesis of these diseases can be summarized as ribosomal dysfunction, the pathophysiological process, clinical manifestations, and regime are not exactly the same 95. More in-depth explorations of the pathogenesis of ribosomal diseases at the molecular and cellular levels may assist us in diagnosing and treating such diseases 6, 96 **(Table 1)**.

Currently, the well-established pathogenesis of ribosomopathies is characterized by haploinsufficiency of ribosomal protein and p53-dependent extensive cell-cycle arrest or apoptosis 94, 97, 98. When mutations occur in the genes encoding ribosomal proteins, the balance of ribosomal protein is impaired, thereby leading to haploinsufficiency. Meanwhile, ribosome production is blocked with the accumulation of free ribosomal components such as RPL23, RPL26, RPL5, and RPL11, which can subsequently bind to Murine Double Minute 2 (MDM2) in forming RP-MDM2 complex. Under normal circumstances, MDM2 can couple with p53 ubiquitinatedly to regulate and maintain the stable state of p53. By the time free or abnormal ribosomal proteins competitively bind to MDM2, p53 will be activated and released into the cytoplasm, followed by growth arrest and even apoptosis, in turn resulting in a series of pathological changes 99.

### Congenital diseases

The conception of ribosomopathies is initially proposed in association with the congenital diseases, and congenital hypoplasia represents a series of diseases accounting for the largest proportion of ribosomopathies 95. To the best of our knowledge, ribosomopathies associated congenital diseases include Diamond-Blackfan anemia, the 5q-syndrome, Schwachman-Diamond syndrome, Dyskeratosis congenita, and Treacher Collins syndrome 100-102. These diseases are consistently characterized by the clinical features, such as bone marrow hematopoietic development disorders, congenital malformations, and an increased risk of progressing into cancer. By re-investigating the pathogenesis at the molecular level, it reveals that each disease possesses the corresponding mutation in gene encoding ribosomal proteins which play roles during the process of ribosomal biogenesis. Mutation of the allele encoding functional ribosomal proteins is demonstrated to render ribosomal protein haploinsufficiency.

Diamond-Blackfan anemia (DBA) refers to a congenital hypoplastic anemia, which is characterized by macrocytic anemia, short stature, craniofacial defects, and thumb abnormalities 103, 104. With the development of genetic diagnosing technology in recent years, Draptchinskaia et al. 105 found that approximately half of DBA patients were accompanied by mutations in gene encoding ribosomal proteins, including *RPS19*, *RPS24*, *RPS17*, *RPL35A*, *RPL5*, and *RPL11*, whereas the clinical manifestations caused by disparate ribosomal gene mutations remained highly diverse. For example, *RPL5* mutation was closely related to craniofacial defects, while patients with *RPL11* mutations were at high risk of developing thumb deformities. Unfortunately, the specific pathological mechanisms of different clinical phenotypes caused by different ribosomal gene mutations are still unclear, which may be associated with the different roles played by ribosomal genes during embryonic development. At present, corticosteroids are still first-line therapeutic agents for the treatment of DBA patients, and the good clinical performance of hematopoietic stem cell transplantation is expected to become a radical remedy in treating DBA.

The 5q-syndrome is an independent subtype of myelodysplastic syndrome (MDS) and is initially described by van den Berghe, and it exerts unique clinical manifestations like macrocytic anemia and hypo lobulated micromegakaryocytes 106. Numerous studies have indicated that the 5q-syndrome may be attributed to the mutation of the *RPS14* gene 107, 108. Haploinsufficiency of *RPS14* is noted to cause substantial reduction in the protein level of RPS14, which can further lead to erythropoiesis disorders. At present, lenalidomide is the most effective drug in treating this disease. Although its mechanism underlying action in 5q syndrome is not fully understood, current studies have confirmed that lenalidomide can enhance differentiation of red blood cell, induce cell death via blocking cell division, and promote p53 degradation. In addition to the above mentioned representative congenital dysplasia diseases, there are other diseases that are closely correlated with the mutations of regulatory molecules or constituent proteins, which are indispensable for the biogenesis of ribosomes. For example, Schwachman-Diamond syndrome, Dyskeratosis congenita, Cartilage hair hypoplasia, Treacher Collins syndrome are associated with defects of *SBDS*, *DKC1*, *RMRP*, and *TCOF1* gene, respectively 109-112.

As the key receptor for mammalian ribophagy, NUFIP1 contains a C2H2 zinc finger domain with a nuclear localization signal. Due to its predominant expression in the brain and central nervous system, potential role of NUFIP-1 in the growth and development has received widespread attention, for which studies have shown that NUFIP1 interacts with fragile X mental retardation protein (FMRP) to participate in the regulation of protein synthesis at synaptic sites. Since the important role of NUFIP1 in synaptic plasticity, NUFIP1 may become a promising candidate gene for screening of mental retardation 113. These results suggested that dysfunction of ribophagy might result in the onset of various congenital diseases, in association with ribosomopathies.

### Neurological diseases

Neurological diseases represent a dysfunctional physiological state induced by damage or interruption of the nervous system, including acute injury, chronic neurodegeneration and others (e.g., infectious diseases of the central or peripheral nervous system). Recent studies suggest that ribosomal dysfunction is more or less associated with the occurrence and progression of neurological diseases, which might be one of the intracellular hallmark and pathogenesis of these diseases.

Stroke is the disease with the highest morbidity and mortality among neurological diseases, pathogenesis of which is closely related to the abnormality of cerebral blood vessels. In light of underlying mechanism(s), stroke can be roughly categorized into ischemic and hemorrhagic strokes. Recent reports have documented that endoplasmic reticulum stress (ERS) is of great significance in the pathogenesis of stroke, and resolving of the excessive activation of ERS in neurons can bring about significant neuroprotective impacts 114, 115. A pre-clinical observation confirmed that upregulation of ribophagy exerted a neuroprotective effect on neonatal ischemia and hypoxia 114. On the one hand, it might ameliorate ERS of neurons after neonatal ischemia and hypoxia by activating the autophagic activity targeting ribosomes. On the other hand, the reduction of ribosomal biosynthesis and protein translation allows neurons to retain sufficient energy to cope with damage. The above studies imply that enhanced ribophagy might eliminate the accumulation of unfolded or misfolded proteins in the ER lumen to alleviate ERS, which is conducive to cell survival under stress and even improves the prognosis of stroke patients.

Alzheimer's disease (AD) represents an age-related progressive and devastating neurodegenerative disorder. Majority of AD patients are characterized by early onset of mild cognitive impairment, and AD is also deemed as the primary cause of dementia among the elderly. Recently, emerging evidences from experimental observations have suggested that AD and mild cognitive impairment patients are generally accompanied by cerebral cortex atrophy, reduction of protein content, and impairment of ribosome function, hinting that the onset and development of AD might be attributed to the ribosomal dysfunction 116, 117. Meanwhile, it is accepted that ribosomal dysfunction along with oxidative stress can markedly hinder protein synthesis and give rise to the generation of abnormal proteins. Correspondingly, subsequent proteomic analysis of the brain tissue from AD patients supports such hypothesis. Rocchio et al. 118 found that the small ribosomal subunit comprised of 33 proteins, in which 3 of them (9%) downregulated in AD patients, while 8 (17.3%) of 46 large subunit proteins diminished. These findings indicate that the modifications in translation may epitomize an early astrocyte-specific upshot in AD pathogenesis. Therefore, maintenance and restoration of protein synthesis by targeting repairment of damaged ribosomes is expected to become a novel strategy for treating AD.

Parkinson's disease (PD) is characterized by tremor, muscle rigidity, and decreased movement, and it is the second most common type of neurodegenerative disease among patients over 65 years old. Researcher noted that the nucleolus volume in dopaminergic neurons form PD patients was significantly altered 119. 18S rRNA, 28S rRNA, and a variety of ribosomal proteins including nucleolar proteins nucleolin and nucleophosmin were revealed profound reduction in the brain tissue of PD patients, especially for the* substantia nigra*, suggesting that nucleolar and ribosomal dysfunction might be one of the hallmarks of PD 120. Vilotti et al. 121 reported that mutations in PARK7/DJ-1 gene altered rRNA biogenesis, and it was regarded as the causative factors of both sporadic and familial PD. Of note, researchers have discovered several new mechanisms with respect to PD pathogenesis, including inhibition of mTOR pathway, activation of oxidative stress, and dysfunction of PARkin interacting substrate (PARIS) pathway 122, 123. PARIS not only interacts with 160-kDa Myb-binding protein 1α, a suppressor in rRNA transcriptional and rRNA editing processes, but also communicates with the components of RNA polymerase I. Since these studies have partially verified the close connection between PD and ribosomal dysfunction, development of drugs in relieving nucleolar stress and restoring ribosomal function might bring about breakthroughs in treating patients with PD.

More recently, issued studies have documented that Huntington's disease (HD) is closely correlated with ribosomal dysfunction 124, 125. HD represents a rare fatal but intractable neurodegenerative disease caused by amplified mutations of Huntington protein exon 1 CAG trinucleotide gene. This mutation leads to the amplification of poly-glutamine sequence at the N-terminal of Huntingtin protein, and its intracellular aggregation can result in HD-related clinical manifestations. For instance, Yang et al. 126 found that essential components of RQC complex, Ltn1, Tae2, and heat shock transcription factor 1 could control and monitor the process of mutated Huntingtin protein aggregation transforming into inclusion bodies by regulating the dynamics of actin cytoskeleton. Conversely, the deficiency of RQC genes Ltn1 or Rqc1 resulted in the accumulation of pathogenic proteins in the nucleus, thereby enhancing their cytotoxicity, which was closely related to the occurrence and progression of HD. Taken together, RQC-Hsf1 regulatory system plays pivotal role in mutated Huntingtin detoxification and may become a novel therapeutic target underlying RQC in treating patients with HD.

In addition to the above-mentioned diseases, inactivation of RQCS caused by mutations in the core components of RQC has been reportedly served as another pathogenesis of neurodegenerative diseases. For example, mutations of listerin and GTPBP2 contributed to neurodegeneration in mice 127, 128. However, the neurodegeneration resulted from these mutations possessed disparate features, and the neuron loss was mainly observed in the spinal cord of listerin mutant mice, whereas neuron loss in GTPBP2 mutant mice predominantly occurred in the cerebral cortex and retina. Likewise, NEMF-deficiency resulted in progressive degeneration of motor neurons, neurogenic muscle atrophy, and movement disorders. Investigation of 7 juvenile neuromuscular disease families confirmed the consistent existence of neuromuscular phenotype of NEMF mutation 129. Similarly, GTPBP2 mutations induced Jabi-Elahi syndrome, which represented a neurodegenerative disease characterized by dystonia, motor and sensory neuropathy, ataxia, as well as cognitive impairment 130. Collectively, these evidences indicate that RQCS might be a key player in protecting neurons from degeneration, dysfunction of which appears to be associated with the development and progression of various neurodegenerative disorders. Therefore, therapeutic strategies specifically targeting RQCS for attenuating ribosomal dysfunction and restoring physiological ribosomal function may be potentially effective and require further interrogation.

### Malignant tumors

Ribosomopathies caused by ribosomal dysfunction mainly exhibit with cellular hypoproliferation, including hematopoietic dysfunction and anemia. Paradoxically, once patients survive form these initial hypoproliferation phases, they would have an increased risk in progressing into a hyper-proliferative cellular state and ultimately developing cancer 131, 132. In this regard, patients with Diamond-Blackfan anemia had a five-fold higher risk of cancer than the general population. Surprisingly, these patients were 30-40 times more likely to develop acute myelogenous leukemia, osteosarcoma or colon cancer in comparison to ordinary people 90. In agreement with these findings, 5q-syndrome patients were at higher risk of complicating acute myeloid leukemia, while X-linked dyskeratosis patients were more prone to have myeloid leukemia and various solid tumors 133, 134.

Except for malignancy originated form hematopoietic system, recent studies have shown that ribosomal dysfunction and mutations in gene encoding ribosomal proteins are critically involved in the pathogenesis of several solid tumors 135, 136. Genetic screening revealed that the *RPL5* was missing or mutated in 11% of glioblastomas, 28% of melanoma, 34% of breast cancer. Approximately 10% of patients with gastric, endometrial, and colorectal cancer were accompanied by protein truncation mediated by repeated mutations in gene *RPL22* 132. Moreover, *RPL23A* amplification and *RPSA* mutation could be detected in 12.5% of uterine cancer patients and 3% stomach cancer patients, respectively 137.

Currently, multiple mechanisms have been proposed to explain the oncogenic pathophysiology of ribosomopathies. Firstly, the mutation or deletion of ribosomal genes can directly give rise to the production of non-functional ribosomes, thereby blocking the translational process. Incorrectly transcribed ribosomal genes are possibly translated into proliferation-promoting and even oncogenic proteins. Secondly, in addition to being indispensable components of ribosomes, some RPs possess certain extra-ribosomal functions, for this, recent studies have noticed that RPs are key partners of some major oncogenes such as tumor protein 53 (TP53) and MYC. For example, RPL11 binds c-MYC in the promoter region, in turn inhibiting C-MYC-dependent transcription. TP53 also plays an indispensable role in monitoring protein translation and can be initiated by ribosomal dysfunction 137. Finally, mutations or deletions of ribosomal genes are attributed to some serious external stimuli. In addition to ribosomal genes, the emergence of additional and rescuing mutations in other genes upon ribosomal dysfunction will also increase significantly 137.

Accumulating evidence has demonstrated that NUFIP-1 appears to be related to tumorigenesis. Large-scale transcriptome sequencing of metastatic neuroblastoma showed a mutation of NUFIP1 gene in tumor cells, in which the expression score of NUFIP-1 mutant alleles was greater than 30%, hinting that these mutant genes might have potential carcinogenic effects 138. Identification of the fusion genes in children with acute lymphoblastic leukemia found that the fusion gene ETV6-NUFIP1 might be involved in the development of this disease 139. These results imply that malfunction of NUFIP-1 might be correlated with the tumorigenesis of various malignancies. Nevertheless, the specific roles of NUFIP1-mediated ribophagy in ontogeny are warranted to be further clarified.

### Other diseases

In addition to the congenital diseases, neurodegenerative diseases, and malignant tumors as mentioned above, several investigators have reported that ribosomal dysfunction is closely related to other types of diseases. For instance, Ro protein, a quality control molecule in the process of ribosome biogenesis, could bind to misfolded 5S rRNA precursors, which was key player involving in a lupus-like syndrome. Upon silence of encoding gene of Ro, mice were more sensitive to ultraviolet light and subsequently developed an autoimmune syndrome that was characterized by anti-ribosomal antibodies, anti-chromatin antibodies, and glomerulonephritis 140. Low-density lipoprotein (LDL) is believed to be one of the risk factors for cardiovascular diseases, and contents of LDL and LDL-receptor are elaborately regulated by the protein convertase subtilisin/kexin type 9 (PCSK9) protein. Pellegrino et al. 141 found that R-IMPP, an effective inhibitor of PCSK9, could selectively bind to human 80S ribosomes and block the synthesis of PCSK9 protein. Diminished expression of PCSK9 resulted in periportally elevated LDL-receptor contents and significantly reduced volume of the free LDL, which effectively prevented the occurrence of cardiovascular diseases. In addition, a study proposed that ribosome binding protein eIF5A could serve as a target for anti-inflammatory drugs, since eIF5A interfered with TNF-mRNA translation by regulating the function of ribosomes during protein synthesis, with a proinflammatory response in the production of cytokines and nitroxide 142. Inflammatory bowel disease was associated with attenuated ribosome biogenesis, as evidenced by decreased levels of ribosomal RNAs 143. Hepatitis C virus could bind with RPs as well as 18SrRNA and targeting this interaction might become a potential remedy for treating patients with hepatitis C 144. Strikingly, Nawa et al. 145 initially proposed that nucleophosmin involved in ribosome biogenesis was related to the onset of sepsis and might act as an alarmin for severe sepsis.

## Conclusions and Prospects

As an indispensable organelle for the maintenance of protein homeostasis, ribosome exerts translational functions elaborately under the supervision of various quality control mechanisms from its biogenesis to degradation. Correspondingly, RQCS is comprised of ribosome biogenesis factors, molecular chaperones, RQC complexes, and ribophagy as well as ubiquitination-dependent degrading pathways, which are capable of monitoring and timely recognizing misassembled ribosomes or wrongly synthesized RPs, thereby specifically degrading them to maintain protein homeostasis. Nevertheless, upon exposure to external or internal unfavorable stimuli, the functions and compositions of ribosomes may be impaired and dysregulated, in turn leading to ribosomal dysfunction. So does RQCS, which can malfunction due to various stresses. Once ribosomal dysfunction is irreversible with the disability of RQCS, cellular fates might be potently affected, thereby contributing to the onset of various ribosome-related diseases, including congenital diseases, neurological diseases, and malignant tumors.

The rapid development of gene diagnosing technology prompts researchers to elucidate the possible mechanisms underlying ribosome-related diseases at the genetic and molecular levels. The current findings confirm the close relationship between ribosomal dysfunction and the development of various human diseases, whereas numerous difficulties exist in this field, which require in-depth explorations. Emerging evidence has demonstrated that structural or functional defects of ribosomes are associated with ribosomopathies, but rare studies interrogate the interaction between dysfunction of RQCS and pathogenesis of human diseases. As the indispensable components of RQCS, ribophagy and RQC are mainly studied in yeast and *in-vitro* experiments, mammal-based researches with underlying translational significances appear to be largely lacking, thus hindering us further understanding the pathophysiological role of RQCS in human diseases. Furthermore, since ribosomal dysfunction induced by ribosome collision has been reportedly correlated with various human diseases, dynamic monitoring of ribosomal function might become a rationale method for predicting the development of diseases. Correspondingly, specific markers reflecting functional status of ribosome are needed to achieve this proposal. More importantly, given the potent effects of RQCS in alleviating ribosomal dysfunction, whether targeting RQCS can become a novel therapeutic strategy in treating ribosomopathies deserve through inquiry.

Although there are currently no clinical studies targeted RQCS-related molecules for the treatment of ribosomopathies, emerging evidence suggests the intrinsic relationship between RQCS dysregulation and the onset of various human diseases. It is noteworthy that the proposal of ribosomal protein haploinsufficiency and the p53-related mechanisms may provide novel therapeutic targets in treating ribosomopathies and other human diseases. Particularly, individualized therapeutic strategy on the basis of modulation of RQCS is expected to possess great potential and prospect. Therefore, strengthening the research on precise mechanisms and key regulatory components of RQCS might render novel insights for exploring the management of various ribosomopathies and ribosome-associated diseases.

## Supplementary Material

Supplementary table.

## Figures and Tables

**Table 1:** Ribosome dysfunction and various human diseases

Diseases		Ribosome dysfunction	Clinical manifestation	Therapeutic regimen	Year, authors, and references
Congenital diseases	Diamond-Blackfan anemia	Mutations in genes such as RPS19, RPS24, RPS17, RPL35A, RPL5, and RPL11	Macrocytic anemia, short stature, craniofacial defects, and thumb abnormalities.	Corticosteroids; red cell transfusions; stem cell transplantation.	1978 Nathan DG 103, 104, 105
	5q-syndrome	Mutation of the RPS14 gene	Macrocytic anemia, hypo lobulated micro megakaryocytes.	Lenalidomide; translation enhancer; L-leucine.	1986 van den Berghe H 106,107,108
	Schwachman-Diamond syndrome	SBDS gene defect	Pancreatic insufficiency and bone marrow dysfunction.	Pancreatic enzymatic replacement; transfusions of packed red blood cells (PRBC) and platelet.	2003 Boocock GR 100, 102, 109
	Dyskeratosis congenita	DKC1 gene defect	The triad of oral leukoplakia, nail dystrophy, and reticular hyperpigmentation.	Surveillance for associated complications; stem cell transplantation.	1999 Mitchell JR 110
	Cartilage hair hypoplasia	RMRP gene defect	Short limbed short stature, hypoplastic hair, defective immunity and erythrogenesis.	Prevention of secondary complications.	2001 Ridanpää M 111
	Treacher Collins syndrome	TCOF1 POLR1D, or POLR1B gene defect	Bilateral and symmetric down slanting palpebral fissures, malar hypoplasia, micrognathia, and external ear abnormalities.	Tailored to the specific needs of each individual; craniofacial reconstruction.	2008 Jones NC 112
	Congenital mental retardation	NUFIP-1	Severe distraction, poor memory, and speech ability.	None.	2007 Caselli R 113
Neurological diseases	Stroke	Disturbance of ribophagy and ERS	Sudden weakness on one face, arm or leg, fainting, confusion, and severe headache.	Thrombolytic therapy; surgery.	2014 Carloni S 114,115
	Alzheimer's disease	Decreases in protein contents and the damage of ribosome function	Dementia, cognitive impairment, and dysfunction in global activities.	Cholinesterase inhibitors; memantine; Ab-directed therapies; tau-directed therapeutics.	2005 Ding Q 116,117,118
	Parkinson's disease	18SrRNA, 28SrRNA	Movement problems such as rigidity, slowness, and tremor.	Levodopa; dopamine agonists; and monoamine oxidase-B (MAO-B) inhibitors; deep brain stimulation; MRI-guided focused ultrasound.	1982 Mann DM 119,120,121
	Huntington's disease	Ltn1, Tae2, and heat shock transcription factor 1 (Hsf1)	Involuntary choric movements, behavioral changes and cognitive impairment.	HTT-targeted therapies; nonselective or allele-selective HTT silencing.	2016 Yang J 124,125,126
	Motor neuron disease	NEMF mutation	Motor neurons degeneration, neurogenic muscle atrophy, and movement disorders.	Respiratory support, nutritional support, symptomatic treatment, drug treatment.	2020 Martin PB 129
	Jabi-Elahi syndrome	GTPBP2 mutation	Dystonia, motor and sensory neuropathy, ataxia, and cognitive impairment.	Symptomatic treatment, drug treatment.	2018 Bertoli-Avella AM 130
Malignant tumors	Acute myelogenous leukemia	TP53 mutations, concomitant with some ribosomopathies	Clonal expansion of abnormally differentiated blasts of myeloid lineage.	Induction therapy; post remission therapy; consolidation chemotherapy with a cytarabine-based regimen.	2016 Orsolic I 131,132,133,134
	Glioblastomas	Mutations or deletion of RPL5 gene	Headache, nausea and vomiting, epilepsy, and blurred vision.	Incorporating surgery, radiotherapy, systemic therapy (chemotherapy, targeted therapy), and supportive care	2018 Pelletier J 132,137
	Melanoma	Mutations or deletion of RPL5 and RPL11 genes	Damaged skin and pigmentation.	Surgical excision and lymph node biopsy; adjuvant treatment.	2019 Sulima SO 137
	Breast cancer	Mutations or deletion of RPL5 and RPL11 genes	Breast lumps, nipple discharge, and skin changes.	Surgery, chemotherapy, radiotherapy, systemic therapy.	2009 Belin S 136,137
	Gastric, endometrial and colorectal cancer	Mutations of the RPL22 gene; RPL23A gene amplification and RPSA gene mutation	Digestive symptoms, and anemia.	Surgery, chemotherapy, radiotherapy, systemic therapy.	2019 Sulima SO 129,137
	Uterine cancer	RPL23A gene amplification and RPSA gene mutation	Painless hematuria, frequent urination, urgency and other urinary symptoms.	Surgery, chemotherapy, radiotherapy, systemic therapy.	2019 Sulima SO 137
	Metastatic neuroblastoma	Mutations of NUFIP-1	Fever, general malaise, weight loss, bone pain, constipation or diarrhea.	Surgery, chemotherapy, radiotherapy.	2018 Wei JS 138
	Acute lymphoblastic leukemia	Mutations of union gene ETV6-NUFIP1	Anemia, fever and infection, bleeding, and organ tissue infiltration.	Hematopoietic stem cell transplantation, induction therapy; post remission therapy; consolidation chemotherapy with a cytarabine-based regimen.	2019 Mata-Rocha M 139
Other diseases	Lupus-like syndrome	Ro protein	An autoimmune syndrome characterized by anti-ribosomal antibodies, anti-chromatin antibodies, and glomerulonephritis.	Promoting the expression of Ro protein, avoiding ultraviolet radiation, immunotherapy.	2003 Xue D 140
	Cardiovascular disease	R-IMPP selectively bind to human 80S ribosomes	Palpitation, shortness of breath, chest tightness, chest pain, fatigue, and edema.	Inhibiting the expression of convertase subtilisin/ kexin type 9 (PCSK9) protein	2016 Pellegrino S 141
	Inflammatory bowel disease	Decline of ribosome biogenesis eIF5A	Abdominal pain, diarrhea, bloody stools, and weight loss.	Targeted regulation of ribosome biogenesis eIF5A.	2016 Figueiredo VC 142,143
	Hepatitis C	Disorder of interaction between ribosomal proteins and 18SrRNA	Nausea, decreased appetite, general weakness, and jaundice.	Targeting the interaction between ribosomal proteins and 18SrRNA.	2018 Bastide A 144
	Severe sepsis	Nucleophosmin	Organ dysfunction, circulatory failure, and shock.	Acting as an alarmin to detect severe sepsis patients early.	2009 Nawa Y 145

Abbreviations: RPs: ribosomal proteins; ERS: endoplasmic reticulum stress; NUFIP-1: nuclear fragile X mental retardation-interacting protein 1; TP53: tumor protein 53.

## Abbreviations

RQCS: ribosome quality control system
RQC: ribosome quality control
rRNA: ribosomal RNA
RPs: ribosomal proteins
snoRNAs: small nucleolar RNAs
LSU: the 60S large subunit
SSU: the 40S small subunit
PTC: peptidyl transferase center
mRNAs: messenger RNAs
tRNAs: transfer RNAs
OXPHOS: oxidative phosphorylation
EIF3: eukaryotic initiation factor 3
mTOR: mammalian target of rapamycin
Ifh1: interacts with forkhead 1
Utp22: U3 small nucleolar RNA-associated protein 22
U3-snoRNP: U3 small nucleolar ribonucleoprotein particles
Efg1: exit of G1
TRAMP: TRf4/5-Air1/2-Mtr4 polyadenylation
Mdn1: midasin AAA ATPase 1
NGD: no-go decay
RACK1: receptor for activated C kinase 1
ERISQ: excess ribosomal protein quality control
UBE2O: ubiquitin conjugating enzyme E2O
NRD: nonfunctional rRNA decay
NUFIP-1: nuclear fragile X mental retardation-interacting protein 1
ZNHIT3: Zinc finger HIT domain containing protein 3
LAMP2C: lysosomal associated membrane protein 2C
CMA: chaperone-mediated autophagy
HSPA8: heat shock 70 kD protein 8
SIDT2: SID-1 transmembrane family member 2
ARM: arginine-rich motif
MDM2: murine double minute 2
DBA: Diamond-Blackfan anemia
MDS: myelodysplastic syndrome
FMRP: fragile X mental retardation protein
ERS: endoplasmic reticulum stress
AD: Alzheimer's disease
PD: Parkinson's disease
PARIS: PARkin interacting substrate
HD: Huntington's disease
TP53: tumor protein 53
LDL: low-density lipoprotein
PCSK9: protein convertase subtilisin/kexin type 9
