# Supplementary material for: Eukaryotic ribosome quality control system: a potential therapeutic target for human diseases
Source: Int J Biol Sci. 2022 Mar 14;18(6):2497–514. doi: 10.7150/ijbs.70955 (PMC8990456; doi:10.7150/ijbs.70955)
Supplement: Supplementary file 1 — Supplementary table. [file ijbsv18p2497s1.pdf]

Supplemental Table 1. Dedicated chaperones and corresponding ribosome proteins

Year	Authors	Dedicated chaperones	Ribosome proteins	References
1997	Eisinger DP	Sqt1	Rpl10	45
2001	Iouk TL	Rrb1	Rpl3	43
2012	Kressler D	Syo1	Rpl1/ Rpl5	46
2012	Koch B	Yar1	Rps3	48
2014	Loc'h J	Fap7	Rps14	49
2014	Schütz S	Tsr2	Rps26	50
2015	Pillet B and Stelter P	Acl4	Rpl4	42,44
2017	Ting YH	Bcp1	Rpl23	41
2019	Liang KJ	Puf6 /Loc1	Rpl43	38
2019	Black JJ and Rössler I	Tsr4	Rps2	39,40
2019	Rössler I	Nap1	Rps6	40
